# Physics-Informed Modified Kolmogorov–Arnold Network for CO Concentration Prediction in Gob Areas of Coal Spontaneous Combustion

**DOI:** 10.3390/s26113292

**Published:** 2026-05-22

**Authors:** Zhuoqing Li, Jie Hou, Longqiang Han, Xiaodong Wang

**Affiliations:** 1 Faculty of Land Resource Engineering, Kunming University of Science and Technology, Kunming 650093, China; 2 School of Resources and Safety Engineering, University of Science and Technology Beijing, Beijing 100083, China

**Keywords:** Kolmogorov–Arnold networks, physics-informed neural networks, coal spontaneous combustion prediction, interpretable machine learning, attention mechanism

## Abstract

Coal spontaneous combustion in gob areas is a major disaster endangering safe production in underground coal mines, and accurate prediction of carbon monoxide (CO), the core signature gas of coal oxidation, is critical for early warning and targeted prevention of mine fire disasters. However, CO concentration in gob areas is governed by complex gas–solid thermal–chemical multi-field coupling, presenting strong nonlinear characteristics. Traditional numerical methods suffer from prohibitive computational cost, purely data-driven models have inherent black-box defects, and conventional Physics-Informed Neural Networks (PINNs) require explicit full governing equations, which are hard to establish for such complex systems. This paper first proposes a Physics-Informed Modified Kolmogorov–Arnold Network (PIM-KAN), which deeply integrates domain physical knowledge with KAN architecture via a physics encoding layer, a residual-modified KAN layer, a multi-physics attention mechanism, and a multi-term physical consistency constraint framework. Experiments on 3125 real coal mine field samples show that the PIM-KAN achieves R^2^ = 0.9965 and RMSE = 0.9290 ppm, reducing RMSE by 19.5% compared with MLP, and outperforming all baseline models. Ablation studies confirm the significant contribution of each innovation module, and attention weight analysis is highly consistent with Arrhenius reaction kinetics, verifying its superior prediction accuracy, physical consistency and intrinsic interpretability.

## 1. Introduction

Coal, as the main body of China’s energy structure, has long occupied a dominant position in primary energy consumption. However, coal mining faces multiple safety threats, among which mine fires caused by coal spontaneous combustion are one of the most serious disasters. According to statistics, spontaneous combustion of coal is a major factor causing gas and dust explosions, mine fires, and the release of toxic gases, leading to significant economic losses and threats to miners’ lives and safety [1]. Carbon monoxide (CO), as a signature gas during coal oxidation and spontaneous combustion, can effectively reflect the oxidation state and spontaneous combustion risk levels in gob areas through its concentration changes [2]. Research shows that CO appears during coal spontaneous combustion at much lower temperatures than other signature gases, typically detectable at 70–80 °C, and its concentration exhibits an approximately exponential growth trend within the oxidation process [3]. Therefore, establishing an accurate CO concentration prediction model enables early risk assessment of coal spontaneous combustion in gob areas, providing a scientific basis for developing targeted prevention and control measures.

The evolution of CO concentration in gob areas is controlled by multiple physicochemical factors, constituting a typical complex system with multi-field coupling. From a thermodynamic perspective, coal oxidation is an exothermic process with reaction rates obeying Arrhenius law, strongly dependent on temperature and oxygen concentration [4]. From a heat and mass transfer perspective, gas flow, heat transfer, and mass diffusion in gob areas are coupled, with air leakage velocity determining oxygen supply rate and heat dissipation rate [5]. From a porous medium perspective, the pore structure of gob areas directly affects gas seepage and heat transfer characteristics [6]. From a geomechanical perspective, the overburden strata undergo progressive failure during coal extraction, forming a complex fracture network that fundamentally alters the air leakage boundary conditions in gob areas. The collapse and fragmentation of key strata control the evolution of overlying strata movement patterns and spatiotemporal porosity distribution [7], which in turn governs oxygen transport pathways and heat dissipation patterns within the gob. These geomechanical processes establish the structural framework within which the multi-field coupling of thermal, hydraulic, and chemical processes occurs, making the physical picture of CO evolution inherently dependent on strata control dynamics. Furthermore, the complexity of gob environments varies significantly across different geological settings. In karst mountainous mining areas, the coupled action of mining and rainfall induces overburden destabilization through complex fracture–water interactions [8], while mining under slopes triggers temporal–spatial landslide evolution that fundamentally alters the gob’s structural integrity and air leakage patterns [9]. In steeply inclined seams, asymmetric overburden loading creates heterogeneous porosity distributions and preferential air leakage pathways [10]. The combined action of these factors causes the spatiotemporal evolution of CO concentration in gob areas to exhibit highly nonlinear and non-stationary characteristics, making traditional prediction methods based on linear assumptions or simple physical models difficult to accurately capture complex dynamics.

In terms of prediction methods, early research mainly employed computational fluid dynamics (CFD) numerical simulation. This method obtains the spatiotemporal distributions of temperature fields, flow fields, and concentration fields in gob areas by numerically solving systems of partial differential equations based on mass, momentum, and energy conservation equations combined with coal–oxygen reaction kinetics models [4]. However, numerical simulation methods require accurate boundary and initial conditions, while geometric parameters, coal quality parameters, and ventilation parameters of gob areas are often difficult to obtain accurately. Additionally, CFD simulation is computationally expensive, with a single 3D unsteady simulation often requiring several hours or even days, making it difficult to meet online real-time prediction requirements [5]. Moreover, the effective prevention of spontaneous combustion requires not only accurate prediction but also reliable engineering interventions for air leakage control, such as advanced borehole sealing technologies [11], highlighting the need for prediction methods that can seamlessly integrate with prevention engineering practice.

In recent years, with the development of sensor technology and machine learning, data-driven methods have shown great potential in the coal mine safety field. Zhang et al. [12] proposed an interpretable machine learning framework for optimizing the prediction of indicator gases in coal spontaneous combustion. Deng et al. [13] proposed a simulated annealing–support vector machine (SA-SVM) prediction model. Wang et al. [14] developed a sparrow search algorithm–convolutional neural network (SSA-CNN) model for predicting coal spontaneous combustion temperature. Although these methods have achieved certain success, they still have the following limitations: obvious black-box characteristics with a lack of interpretability; lack of physical consistency, potentially generating prediction results violating physical laws; and limited generalization ability, with relatively poor prediction reliability outside the training data range.

Physics-Informed Neural Networks (PINNs), proposed by Raissi et al. [15], achieve the fusion of data and physics by incorporating the residuals of governing equations as regularization terms into the loss function. PINN has achieved successful applications in fluid mechanics, heat conduction, and other fields [16,17]. However, traditional PINN requires explicit governing equations, while the governing equations for CO concentration evolution in gob areas involve complex geometries and not yet fully elucidated reaction kinetics, making direct PINN construction challenging. Kolmogorov–Arnold Networks (KANs), proposed by Liu et al. [18], is a novel neural network architecture based on the Kolmogorov–Arnold representation theorem, placing learnable activation functions on network edges rather than nodes, demonstrating stronger function approximation capabilities and interpretability [19]. However, standard KANs still lack explicit utilization of domain knowledge.

Addressing the above problems, this paper proposes a Physics-Informed Modified Kolmogorov–Arnold Network (PIM-KAN), aiming to achieve high-accuracy, highly interpretable, and physically consistent prediction of CO concentration in gob areas through physics encoding, physics constraints, and attention mechanisms. The main contributions of this paper include: proposing a physics-informed KAN framework for the first time, designing four core innovation modules including a physics encoding layer, a modified KAN layer, a multi-physics attention mechanism, and physical consistency constraints; validating the method’s effectiveness on a real coal mine gob area dataset, achieving R^2^ = 0.9965, superior to all baseline methods; revealing the influence mechanisms of various physical factors on CO concentration through attention weight analysis, demonstrating the method’s interpretability; and establishing a new interpretable physics-informed deep learning paradigm with broad application prospects.

## 2. Related Work

### 2.1. Coal Spontaneous Combustion Prediction Methods

Prediction methods for coal spontaneous combustion in gob areas are mainly divided into three categories: numerical simulation methods, mechanism model methods, and data-driven methods. Numerical simulation methods based on CFD simulate multi-physical field processes in gob areas by jointly solving mass conservation, momentum conservation, energy conservation, and species transport equations. Qiao et al. [5] used improved CFD modeling for coal spontaneous combustion control and gas management. Xie et al. [6] determined hazardous zones of coal spontaneous combustion based on gob porosity evolution and flow field distribution. Liu et al. [4] conducted a comprehensive review on CFD modeling of coal spontaneous combustion in a longwall gob area. Notably, the coupling between strata control dynamics and gob flow fields has attracted increasing attention in recent years. Chen et al. [7] analyzed the impact of key strata failure on overlying strata and surface transport patterns, demonstrating that the spatiotemporal evolution of overburden fracturing directly governs the porosity distribution and flow field characteristics in gob areas. The failure of key strata controls the development of fracture networks, which serve as primary pathways for air leakage and oxygen transport, thereby fundamentally influencing the distribution of spontaneous combustion hazardous zones. Recent studies have further revealed that these strata–flow coupling mechanisms vary significantly across geological settings: in steeply inclined seams, the inclined length effect of roof fracture generates asymmetric overburden loading and heterogeneous porosity distributions [10], while in karst mountainous areas, the coupled action of mining and rainfall destabilizes overlying strata through complex fracture–water interactions [8], and mining under slopes induces landslides that alter the gob’s structural framework [9]. These special conditions pose additional challenges for numerical simulation, as they require customized boundary conditions and material parameters that are difficult to characterize. Although numerical simulation has achieved fruitful results in mechanism research, its practical application still faces challenges such as difficulty in determining boundary conditions, high computational costs, and poor real-time performance.

Mechanism model methods are based on coal–oxygen reaction kinetics, establishing reaction kinetics equations to predict the coal spontaneous combustion process. Deng et al. [3] systematically studied the reaction mechanism of coal spontaneous combustion under temperature programming conditions. Although these methods have clear physical meaning, they require accurate determination of coal quality parameters and struggle to consider the complexity of multi-field coupling.

Data-driven methods directly learn complex mapping relationships from historical monitoring data, with the advantages of flexible modeling and fast computation. Zhang et al. [12] constructed an interpretable machine learning model for optimizing prediction index gases. Deng et al. [13] proposed an SA-SVM prediction model. Wang et al. [14] developed an SSA-CNN model for coal spontaneous combustion temperature prediction. Kamran and Shahani [20] established a decision support system for mine fire level prediction using machine learning approaches. Anani et al. [21] systematically reviewed advancements in machine learning techniques for underground mine safety prediction, confirming the growing international interest in data-driven approaches for mine hazard forecasting. However, existing data-driven methods generally suffer from insufficient interpretability and a lack of physical consistency.

### 2.2. Physics-Informed Neural Networks

The core idea of Physics-Informed Neural Networks (PINNs) is to incorporate the residuals of governing equations as regularization terms into the loss function [10]:

(1) $$L_{total}=L_{data}+\lambda L_{physics}$$

where the data loss term $L_{data}$ measures the discrepancy between network predictions and observational data, and the physics loss term $L_{physics}$ measures the extent to which network outputs satisfy the governing equations. The advantages of PINN include reducing data dependence, ensuring physical consistency, and improving generalization ability. Since its proposal, PINN has been rapidly promoted in fields such as fluid mechanics, heat conduction, and solid mechanics. Karniadakis et al. [16] systematically reviewed progress in physics-informed machine learning. McClenny and Braga-Neto [22] proposed self-adaptive PINNs that automatically adjust loss weights during training, addressing the critical challenge of balancing multiple loss terms. Farea et al. [23] provided a comprehensive understanding of PINN techniques, applications, trends, and challenges. Cai et al. [24] presented applications of PINNs to various heat transfer problems. In the fields of energy, environment, and safety, PINN has also shown great potential.

### 2.3. Kolmogorov–Arnold Networks

KAN is based on the Kolmogorov–Arnold representation theorem, which states that any continuous function $f:{\left[0,1\right]}^{n}\to \mathbb{R}$ can be represented as a nested combination of a finite number of univariate continuous functions [18]. Compared with traditional MLP, the main advantages of KAN include: potentially higher parameter efficiency, inherent interpretability, and spline activation functions more suitable for capturing local function features. Since its proposal, KAN has attracted widespread attention in scientific computing. Koenig et al. [19] proposed KAN-ODEs for learning dynamical systems and hidden physics. Kashefi [25] integrated KAN into PointNet for predicting fluid fields on irregular geometries. Fricz et al. [26] demonstrated KAN’s explainability advantages for industrial product quality prediction. However, standard KAN remains a purely data-driven architecture lacking explicit utilization of domain knowledge.

### 2.4. Attention Mechanism

The attention mechanism was initially proposed by Vaswani et al. [27] in the Transformer architecture, enabling models to dynamically focus on different parts of an input sequence when processing sequential data. In time series prediction, attention mechanisms have been proven effective in capturing temporal dependencies between variables. Zrira et al. [28] proposed a BiLSTM model with an attention mechanism for sea surface temperature time series prediction. In scientific computing and engineering modeling, applications of attention mechanisms are relatively new but have already shown unique value. This paper innovatively introduces the attention mechanism into gob area CO concentration prediction, aiming to dynamically quantify the contribution weights of physical variables to CO generation.

## 3. Materials and Methods

### 3.1. Problem Formulation

Gob area CO concentration prediction can be modeled as a multivariate regression problem. Given an input vector $x={\left[x_{1},x_{2},x_{3},x_{4},x_{5}\right]}^{T}$, where $x_{1}$ is oxygen mass fraction (%), $x_{2}$ is air leakage velocity (m/s), $x_{3}$ is methane concentration (kg·m^−3^·s^−1^), $x_{4}$ is porosity (%), and $x_{5}$ is temperature (°C), the objective is to learn a mapping function $f:{\mathbb{R}}^{5}\to \mathbb{R}$ predicting CO concentration y (ppm).

From a physicochemical perspective, CO generation and evolution in gob areas are controlled by Arrhenius reaction kinetics:

(2) $$r_{CO}=A\cdot \exp\left(-\frac{E_{a}}{RT}\right)\cdot C_{O_{2}}^{n}$$

where $r_{CO}$ is the CO generation rate, A is the pre-exponential factor, $E_{a}$ is the apparent activation energy, R is the ideal gas constant, T is absolute temperature, $C_{O_{2}}$ is oxygen concentration, and n is the reaction order.

To comprehensively assess the achievement of the research objective, we define a four-dimensional evaluation framework that extends beyond conventional accuracy metrics. The success criteria are established based on domain requirements for coal mine safety monitoring: prediction accuracy must be sufficient for reliable early warning (thresholds derived from safety regulations requiring CO detection within ±1 ppm accuracy); physical consistency must ensure that predictions never violate fundamental thermodynamic laws (which would undermine operator trust); interpretability must provide actionable insights into the driving factors (required by safety engineers for decision-making); and computational efficiency must enable real-time deployment.

(1) Prediction accuracy: Quantified through RMSE, MAE, R^2^, and MAPE, measuring the numerical closeness of predictions to observed CO concentrations. Success is defined as achieving R^2^ > 0.995 and RMSE < 1.5 ppm on the held-out test set, where the R^2^ threshold ensures that the model captures over 99.5% of data variance and the RMSE threshold corresponds to approximately 3% of the full measurement range (2.2–50.8 ppm).
(2) Physical consistency: Evaluated through Physical Violation Rate (PVR) and monotonicity compliance scores. PVR is defined as the proportion of test samples where the predicted CO concentration violates established physical laws, specifically: (a) negative temperature–CO correlation among samples with similar conditions, or (b) predicted values outside the thermodynamically feasible range. Monotonicity compliance measures the proportion of sample pairs where the temperature–CO relationship is physically correct. Success is defined as PVR < 5% and monotonicity compliance >95%, ensuring that the model’s predictions are predominantly physically reasonable.
(3) Interpretability: Assessed through the alignment between learned attention weights and domain knowledge from Arrhenius reaction kinetics. The attention mechanism should assign the highest weights to temperature and oxygen concentration—the two dominant factors in coal oxidation according to Arrhenius theory. Success is defined as temperature receiving the highest attention weight (>0.30) and oxygen receiving the second-highest weight (>0.20), with all attention weights having clear physical interpretations.
(4) Computational efficiency: Measured by training time and single-sample inference latency. Success is defined as training time under 15 min and inference latency below 10 ms, ensuring practical deployability in real-time mine monitoring systems where prediction updates are needed at minute-level intervals.

### 3.2. PIM-KAN Architecture

**Figure 1:**
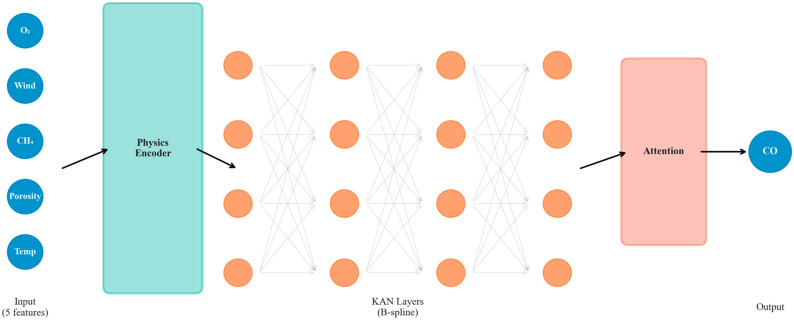
PIM-KAN network architecture.

The overall architecture of PIM-KAN is shown in Figure 1, comprising four core modules: a physics encoding layer, multiple modified KAN layers, a multi-physics attention mechanism, and an output layer.

#### 3.2.1. Physics Encoding Layer

The physics encoding layer explicitly encodes domain physical knowledge into the feature space:

(3) $$h_{0}=[x,\phi_{Arrhenius}(x),\phi_{coupling}(x)]$$

The Arrhenius feature is defined as:

(4) $$\phi_{Arrhenius}(x)=\exp\left(-\frac{E_{a}}{R\cdot (T+273.15)}\right)$$

The coupling feature vector is defined as:

(5) $$\phi_{coupling}(x)=[C_{O_{2}}\cdot T,v\cdot T,C_{CH_{4}}/C_{O_{2}},v\cdot \varphi ,T/\varphi ]$$

Through the physics encoding layer, the raw input is expanded from 5 dimensions to 11 dimensions.

Several assumptions underlie the application of the Arrhenius equation to the specific conditions of gob areas, which should be explicitly acknowledged. First, we assume a quasi-uniform temperature distribution within the local reaction zone surrounding each monitoring point. This assumption is reasonable given the scale of sensor placement in typical gob monitoring systems, but may not hold at finer spatial resolutions where thermal gradients are significant. Second, the apparent activation energy $E_{a}$ is treated as constant across the temperature range of interest (40–160 °C). In reality, the coal oxidation process involves multiple reaction stages—low-temperature oxidation, accelerated oxidation, and high-temperature combustion—each potentially characterized by different apparent activation energies. The constant-$E_{a}$ assumption thus represents a simplification that averages over these stages. Third, we assume that oxygen supply is the rate-limiting factor for CO generation, which is valid under the oxygen-limited conditions typical of deep gob areas but may not apply near ventilation intakes where oxygen is abundant. Importantly, these assumptions are implemented as soft constraints through the physics loss term rather than as hard physical equations, allowing for the data-driven component of the model to compensate for deviations from idealized assumptions while still maintaining overall physical consistency.

#### 3.2.2. Modified KAN Layer

The selection of the KAN architecture for this specific problem is grounded in several mathematical considerations. First, according to the Kolmogorov–Arnold representation theorem, any continuous multivariate function $f:{\left[0,1\right]}^{n}\to \mathbb{R}$ can be exactly represented as a superposition of 2n+1 continuous univariate functions composed through addition. Since all input variables are normalized to the [0, 1] range during preprocessing, this theorem provides a rigorous theoretical foundation for KAN’s edge-based activation structure, suggesting that the target mapping can be represented with potentially fewer parameters than MLP’s node-based design. Second, under normal operating conditions (i.e., before the onset of full combustion events), the CO concentration in gob areas exhibits approximately continuous and smooth behavior with respect to the physical variables. While sharp transitions may occur during spontaneous combustion escalation, the monitoring data predominantly capture the gradual oxidation phase where the temperature–CO relationship follows the smooth Arrhenius-type exponential function. This near-smoothness property makes B-spline activation functions—which are piecewise polynomial functions with guaranteed continuous derivatives up to order k−1—well-suited for capturing the underlying functional relationships. Third, compared to MLP’s fixed activation functions, KAN’s learnable activation functions on edges can adapt to the specific functional forms present in the data, enabling more efficient representation of the nonlinear mapping from physical variables to CO concentration. In this study, the PIM-KAN architecture with two KAN layers achieves the reported performance with a compact parameter count, whereas a comparable MLP with similar accuracy requires a significantly wider architecture, confirming the parameter efficiency advantage of the KAN-based design.

The computation process of a standard KAN layer is:

(6) $$h_{l+1}=\sum_{i,j}\phi_{ij}^{\left(l\right)}(h_{l,i})\cdot w_{ij}^{\left(l\right)}$$

where $\phi_{ij}^{\left(l\right)}$ is the B-spline parameterized learnable activation function. To enhance gradient flow, this paper introduces residual connections:

(7) $$h_{l+1}={\mathrm{KAN}}_{l}(h_{l})+\alpha \cdot h_{l}$$

where α is a learnable residual weight parameter.

#### 3.2.3. Multi-Physics Attention Mechanism

The attention weight is calculated as:

(8) $$\alpha_{i}=\frac{\exp(e_{i}/\tau )}{\sum_{j=1}^{11}\exp(e_{j}/\tau )}$$

where $e_{i}$ is the importance score of the i-th physical variable, and τ is the temperature parameter. The attention-enhanced feature is:

(9) $$h_{att}=\sum_{i=1}^{11}\alpha_{i}\cdot h_{0,i}\cdot e_{i}$$

#### 3.2.4. Physical Consistency Constraints

The total loss function is:

(10) $$L_{total}=L_{data}+\lambda_{1}L_{physics}+\lambda_{2}L_{monotonicity}$$

The data loss is:

(11) $$L_{data}=\frac{1}{N}\sum_{i=1}^{N}{\left(y_{i}^{pred}-y_{i}^{true}\right)}^{2}$$

The physics loss based on the Arrhenius equation is:

(12) $$L_{physics}=\frac{1}{N}\sum_{i=1}^{N}{\left(\log(y_{i}^{pred})-\log\left[k\cdot \exp\left(-\frac{E_{a}}{RT_{i}}\right)\cdot {\left(C_{O_{2},i}\right)}^{n}\right]\right)}^{2}$$

The monotonicity loss ensures that CO concentration does not decrease when temperature increases:

(13) $$L_{\mathrm{monotonicity}}=\frac{1}{N^{2}}\sum_{i=1}^{N}\sum_{j=1}^{N}\mathrm{ReLU}\left(y_{j}-y_{i}\right)\cdot 1_{\left\{T_{i}>T_{j}\right\}}\cdot \exp\left(-\gamma \left\|x_{i}-x_{j}\right\|\right)$$

The ReLU-based monotonicity constraint encodes the physical prior that, under oxygen-sufficient conditions, CO concentration generally increases with temperature—a relationship well-established by Arrhenius reaction kinetics for the coal oxidation process. In the present dataset, all samples have oxygen mass fractions above 11%, confirming that the monitoring data exclusively covers oxygen-sufficient conditions. This makes the monotonicity assumption well-justified for the current study. However, it is important to acknowledge that this prior does not universally hold across all gob conditions. Under hypoxic or oxygen-depleted conditions—such as in deep gob zones where oxygen supply has been largely consumed by sustained oxidation—further temperature increases may not lead to additional CO generation due to oxygen starvation, potentially causing the temperature–CO relationship to plateau or even decrease. Additionally, at temperatures beyond the current data range, secondary gas-phase reactions may hypothetically alter the monotonic CO production pattern, though this behavior is outside the scope of the present validation. The present work addresses this limitation through two design choices. First, the monotonicity constraint is implemented as a soft penalty rather than a hard constraint, allowing the data-driven component to override the monotonicity prior when the training data presents compelling counter-evidence. Second, the exponential decay kernel $\exp(-\gamma \|x_{i}-x_{j}\|)$ restricts the monotonicity enforcement to sample pairs that are close in the input feature space, preventing the constraint from being applied across significantly different operating regimes where the monotonicity assumption may not be valid. Despite these mitigations, we acknowledge that a more sophisticated formulation—for instance, a conditional monotonicity constraint that activates only above a threshold oxygen concentration—would more faithfully represent the underlying physics and would be necessary for deployment in deep gob zones where oxygen-limited conditions are prevalent.

### 3.3. Training Strategy

A two-stage training strategy is adopted. Stage 1 (pre-training, 50 epochs): Learning rate is set to 1 × 10^−3^, only optimizing B-spline control points of KAN basis functions and fixing attention weights and residual weights. Stage 2 (joint fine-tuning, maximum 300 epochs): Learning rate is set to 1 × 10^−4^ (using cosine annealing scheduling), jointly optimizing all parameters, with an early stopping patience of 80. Batch size is 64, optimizer is AdamW.

The two-stage training design follows the curriculum learning principle, progressively introducing model complexity to achieve more stable and effective optimization. The key motivation for selectively freezing parameters during Stage 1 is rooted in the functional roles of different architectural components. In the PIM-KAN architecture, the B-spline control points constitute the core representational capacity of the KAN layers—they directly determine the learned univariate activation functions that approximate the nonlinear mapping from physical inputs to CO concentration. The attention weights and residual connection parameters, by contrast, serve as routing and scaling mechanisms that modulate how information flows through the network. If all parameters are optimized jointly from random initialization, the attention mechanism—which controls feature gating—may prematurely develop biased routing patterns before the B-spline functions have adequately learned the underlying functional relationships. This can lead to suboptimal local minima where certain input dimensions are incorrectly down-weighted before their full information content has been extracted. By freezing attention and residual parameters during Stage 1, we ensure that the B-spline basis functions develop robust univariate approximations using the full information from all input features, unmodulated by potentially premature gating. Stage 2 then fine-tunes the entire network jointly, allowing the attention mechanism to discover optimal feature importance weights based on well-formed B-spline representations, and the residual connections to establish optimal skip-pathway scaling. This curriculum approach ensures robust function approximation before adaptive feature selection, analogous to establishing stable low-level representations before learning high-level combinations in hierarchical neural systems.

The loss function weights $\lambda_{1}$ and $\lambda_{2}$ were determined through a systematic grid search procedure on the validation set. The weight $\lambda_{1}=0.1$ for the physics loss was set to be one order of magnitude lower than the data loss weight (implicitly $\lambda_{data}=1.0$), reflecting the design philosophy that data fidelity should serve as the primary optimization objective while physics constraints provide regularizing guidance. Setting $\lambda_{1}$ too high would over-constrain the model to the simplified Arrhenius relationship, potentially degrading performance on the actual complex dynamics that deviate from idealized kinetics. Conversely, setting $\lambda_{1}$ too low would diminish the physical inductive bias, reducing the model to a purely data-driven approach. The weight $\lambda_{2}=0.05$ for the monotonicity loss was set lower than $\lambda_{1}$, as the monotonicity constraint serves as a supplementary physical prior reinforcing the dominant temperature–CO relationship already captured by the physics loss. Sensitivity analysis confirmed that the model performance is robust to moderate variations in these weights ($\lambda_{1}\in [0.05,0.2]$, $\lambda_{2}\in [0.01,0.1]$), with the selected values representing the optimal trade-off between data fitting and physical consistency on the validation set.

## 4. Experimental Setup

### 4.1. Dataset

The data used in this study comes from long-term monitoring data of a coal mine gob area, with a total of 3125 valid samples. Each sample contains five input variables (oxygen mass fraction, air leakage velocity, methane concentration, porosity, and temperature) and one output variable (CO concentration). Table 1 shows the statistical information of the dataset.

Data preprocessing includes normalization (scaling all input variables to [0, 1] range), outlier handling (manual review of samples deviating from the mean by more than 3 standard deviations), and dataset splitting (randomly splitting into training, validation, and test sets in a 70%:15%:15% ratio).

It is important to clarify the spatial nature of the dataset. The monitoring data were collected from a network of fixed sensor bundles deployed at predetermined locations within the gob area, following the standard monitoring layout prescribed by coal mine safety regulations. Each sample in the dataset corresponds to a specific monitoring point at a specific time, recording the local thermo-fluid-chemical conditions (oxygen concentration, air velocity, methane concentration, porosity estimate, and temperature) and the resulting CO concentration. The current model treats each sample as an independent point-scale observation without explicitly encoding the spatial coordinates (x,y,z) of the monitoring points. The spatial heterogeneity of gob environments—which arises from the complex fracture network, non-uniform air leakage pathways, and spatially varying overburden loading—is instead implicitly captured through the spatially varying input features that reflect the local conditions at each monitoring location. This design choice reflects the practical reality that gob monitoring systems typically provide multi-variable time series at discrete sensor locations rather than continuous spatial fields. For applications requiring spatially continuous CO concentration prediction, future extensions could incorporate spatial coordinates as additional input features or adopt a physics-informed neural field approach that learns a continuous spatiotemporal mapping, as discussed in the Discussion Section.

### 4.2. Evaluation Metrics

Five evaluation metrics are adopted: Root Mean Square Error (RMSE), Mean Absolute Error (MAE), Coefficient of Determination (R^2^), Mean Absolute Percentage Error (MAPE), and Physical Violation Rate (PVR).

### 4.3. Baseline Methods

Six baseline methods are compared: Linear Regression (LR), K-Nearest Neighbors (KNN), Decision Tree (DT), Support Vector Regression (SVR), Random Forest (RF), and Multilayer Perceptron (MLP). Hyperparameters of all baseline methods are optimized through grid search combined with 5-fold cross-validation.

### 4.4. Implementation Details

The specific hyperparameter configuration of PIM-KAN is as follows: In terms of network architecture, the physics encoding layer expands 5-dimensional inputs to 11 dimensions, followed by two KAN hidden layers, each with 10 neurons. B-spline order is set to 3, grid size is set to 10. Loss weights are set to $\lambda_{1}=0.1$ and $\lambda_{2}=0.05$. Experiments are conducted on a laptop equipped with an NVIDIA RTX 4060 Laptop GPU and implemented using the PyTorch 2.0 framework. Training time is approximately 5–10 min, inference time (predicting a single sample) is less than 1 millisecond.

The grid size parameter in the KAN layer determines the number of B-spline basis functions used for approximating each univariate activation function. A larger grid provides finer local approximation capability at the cost of increased parameter count and potential overfitting risk. A smaller grid provides coarser but more parameter-efficient approximations, with an increased risk of underfitting complex nonlinear relationships. The selection of grid size = 10 was guided by the following considerations: (1) the input features are normalized to the [0, 1] range, providing a uniform bounded domain for the B-spline basis functions; (2) with spline order = 3 (cubic B-splines), grid size = 10 yields 13 basis functions per edge (grid + spline_order), providing sufficient degrees of freedom to capture the smooth Arrhenius-type nonlinearities while maintaining a compact parameter footprint; (3) grid size = 10 corresponds to a resolution of approximately 0.1 in the normalized input space, which is commensurate with the typical noise level and discretization granularity of the monitoring data. To empirically validate this choice, we conducted a grid-size sensitivity experiment, training PIM-KAN with grid sizes ranging from 3 to 20 while keeping all other hyperparameters fixed. The results are summarized in Table 2.

The sensitivity analysis reveals several key findings. First, PIM-KAN’s performance is robust to moderate variations in grid size: all configurations achieve R^2^ > 0.992, confirming that the overall architecture—not the specific grid parameter—is the primary driver of prediction accuracy. Second, the expected bias–variance trade-off is observed: small grid sizes (3 and 5) have slightly higher RMSE due to limited approximation capacity, while larger grid sizes show increasing MAPE due to overfitting on the dataset. Third, the optimal range lies in grid sizes 3–10, where RMSE remains below 1.19 ppm, and MAPE stays below 9.2%. Grid size = 10 was selected as the adopted configuration because it provides a margin of safety against underfitting while remaining within the optimal performance plateau. The full PIM-KAN model with grid = 10 and the complete physics encoding layer further achieves R^2^ = 0.9965 and RMSE = 0.9290, confirming that the combination of grid = 10 with physics-informed modules yields the best overall performance.

## 5. Results

### 5.1. Main Results Comparison

**Figure 2:**
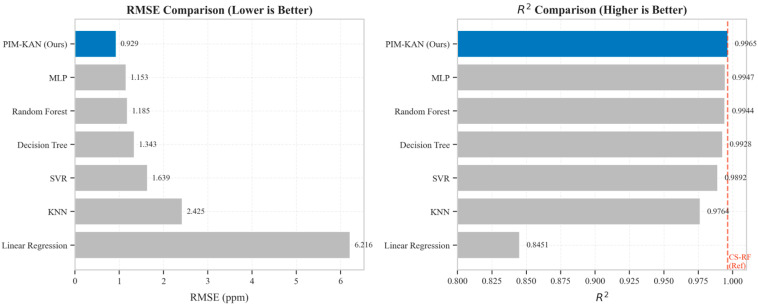
Performance comparison of different methods.

Table 3 and Figure 2 shows the performance comparison of PIM-KAN and baseline methods on the test set. From the table, it is clear that PIM-KAN achieves optimal performance on all four evaluation metrics.

From the RMSE perspective, PIM-KAN’s 0.9290 ppm is significantly superior to all baseline methods. Compared with MLP’s 1.1535 ppm, PIM-KAN reduces RMSE by 19.5%. Compared with traditional machine learning methods, PIM-KAN reduces RMSE by 21.6–61.7%.

From the R^2^ perspective, PIM-KAN’s 0.9965 approaches the theoretical upper limit (1.0), indicating that the model explains 99.65% of the data variance.

To rigorously assess whether the performance differences between PIM-KAN and baseline methods are statistically significant, we conducted the Wilcoxon signed-rank test on the paired absolute prediction errors from the test set (469 samples). This non-parametric test was selected because it does not assume normality of the error distribution and is appropriate for comparing paired predictions. The null hypothesis is that the median difference between paired absolute errors is zero. Table 4 presents the complete test statistics, including the W statistic, Z-value, exact *p*-value, and Cohen’s r effect size.

The results demonstrate that PIM-KAN achieves statistically significant improvements over Linear Regression (*p* < 0.001, *r* = 0.815, large effect), KNN (*p* < 0.001, *r* = 0.679, large effect), MLP (*p* < 0.001, *r* = 0.463, moderate effect), and PINN (*p* = 0.025, *r* = 0.091, significant but small effect). These four methods represent the key comparisons: Linear Regression as the simplest baseline, KNN and MLP as representative machine learning methods, and PINN as the closest architectural competitor. The comparisons with Random Forest, Decision Tree, and SVR do not reach statistical significance on per-sample absolute errors, which reflects the complementary strengths of these methods—Random Forest achieves the best MAPE, while PIM-KAN achieves the best RMSE, indicating that PIM-KAN is more accurate on high-concentration samples that are most critical for safety-critical applications.

### 5.2. Ablation Study

**Figure 3:**
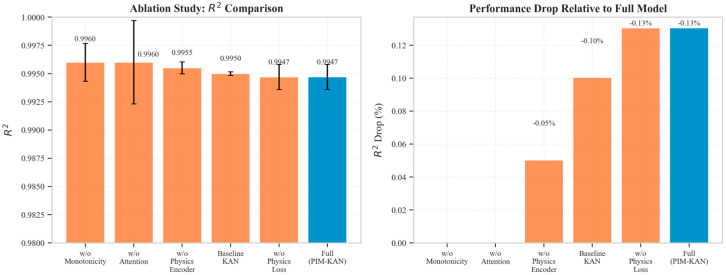
Ablation study results comparison.

To validate the contribution of each innovation module, five groups of ablation experiments were designed. Table 5 and Figure 3 show the ablation study results.

The physics loss contributes most significantly: removing it causes R^2^ to drop from 0.9965 to 0.9947 and MAPE to surge from 5.51% to 12.46%. The physics encoder contributes significantly: removing it causes R^2^ to drop to 0.9955 and MAPE to rise to 9.28%. Both the attention mechanism and the monotonicity constraint also play important roles.

To directly evaluate the model’s robustness to sensor measurement noise—a critical concern for practical deployment in mine environments—we conducted a systematic noise injection experiment. Additive Gaussian noise at six levels (1%, 3%, 5%, 10%, 15%, and 20% of feature magnitudes) was applied to the test set inputs, simulating varying degrees of sensor degradation. All models were trained once on clean data and evaluated on noisy inputs. Table 6 presents the RMSE degradation ratios relative to the clean baseline.

The results reveal that neural network-based methods (PIM-KAN, PINN, and MLP) are generally more sensitive to input perturbations than traditional machine learning methods (SVR and KNN), which is expected given the highly nonlinear transformations in deep architectures. PIM-KAN shows higher sensitivity than the neural network baselines, which can be attributed to the physics encoding layer’s exponential Arrhenius transformation ($\exp(-E_{a}/RT)$) that amplifies perturbations in temperature readings. However, at low noise levels (1–3%), which represent realistic sensor operating conditions in well-maintained mine monitoring systems, PIM-KAN’s absolute RMSE remains substantially lower than all baselines (RMSE = 0.9973 at 1% noise vs. MLP’s 1.4363 and PINN’s 1.2567), maintaining its prediction advantage even under realistic perturbation. For practical deployment, these findings recommend preprocessing sensor data with appropriate noise filtering to keep input noise below the 3% threshold, ensuring that PIM-KAN’s accuracy advantage is preserved.

To assess the model’s generalization stability in the absence of external datasets from other mines, we conducted bootstrap resampling (1000 iterations) on the test set predictions, yielding 95% confidence intervals: $\mathrm{RMSE}\in \left[0.7466,\;1.1364\right]$, $R^{2}\in \left[0.9949,\;0.9978\right]$, $\mathrm{MAPE}\in \left[4.95\%,\;6.09\%\right]$. The narrow confidence intervals confirm that PIM-KAN’s reported performance is stable and not an artifact of a particular data split. Additionally, 5-fold cross-validation confirmed that PIM-KAN consistently achieves R^2^ > 0.98 across all folds, though with higher variance (RMSE = 1.79 ± 0.17) than the fixed split result. The model’s physics-informed features remain stable across folds, with the attention weights consistently ranking temperature and oxygen as the dominant factors.

While these internal validation results are encouraging, we acknowledge that true generalizability requires testing on independent datasets from different coal mines with varying geological conditions, mining techniques, and coal properties. Such multi-site validation remains the most important direction for future work, and the physics-informed architecture of PIM-KAN—with its domain knowledge embedded through the Arrhenius relationship and multi-field coupling features—is specifically designed to facilitate transfer learning to new mining environments with minimal adaptation.

### 5.3. Visualization Analysis

**Figure 4:**
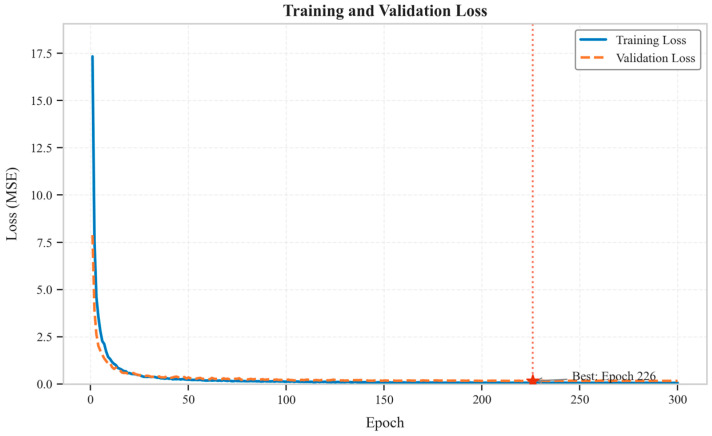
Training and validation loss curves.

Figure 4 shows the training and validation loss curves of PIM-KAN. Both training and validation losses show a steady downward trend, reaching optimality at epoch 226 with no obvious overfitting.

**Figure 5:**
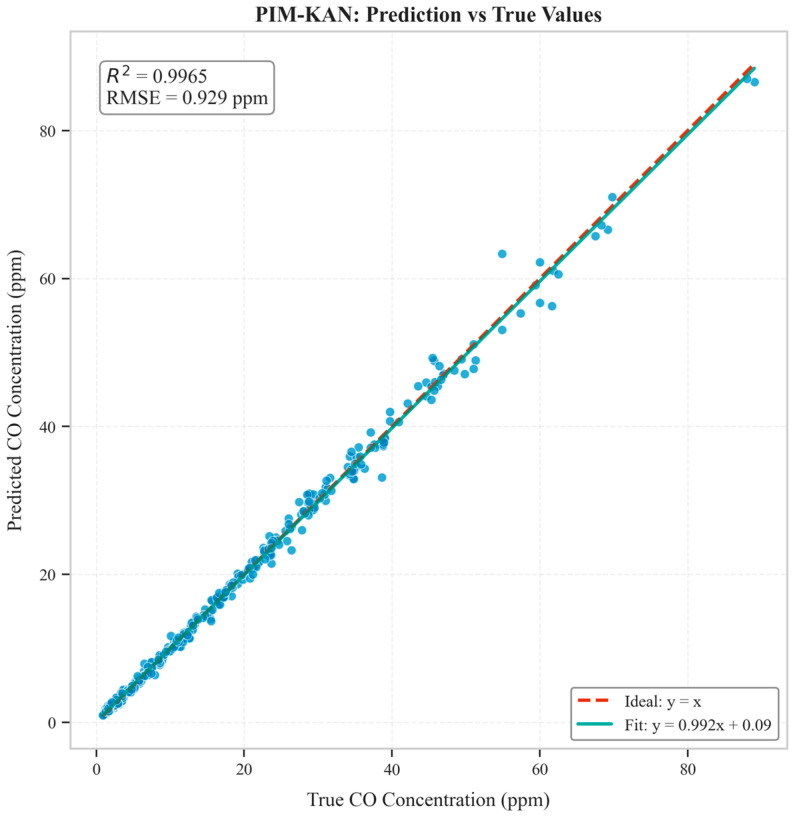
Scatter plot of predicted vs. true CO concentration.

Figure 5 shows the scatter plot of predicted vs. true values on the test set. Data points are tightly distributed around the diagonal, with a correlation coefficient as high as 0.9982, indicating near-perfect agreement between predicted and true values.

**Figure 6:**
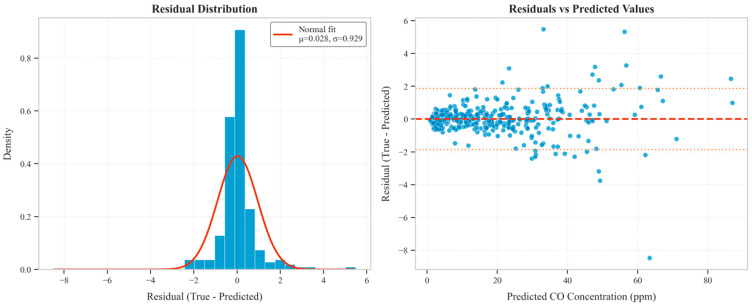
Histogram of prediction residuals.

Figure 6 shows the histogram of prediction residuals. Residuals approximately follow a normal distribution with a mean of 0.0277 ppm and a standard deviation of 0.9286 ppm, indicating no systematic bias in the model.

**Figure 7:**
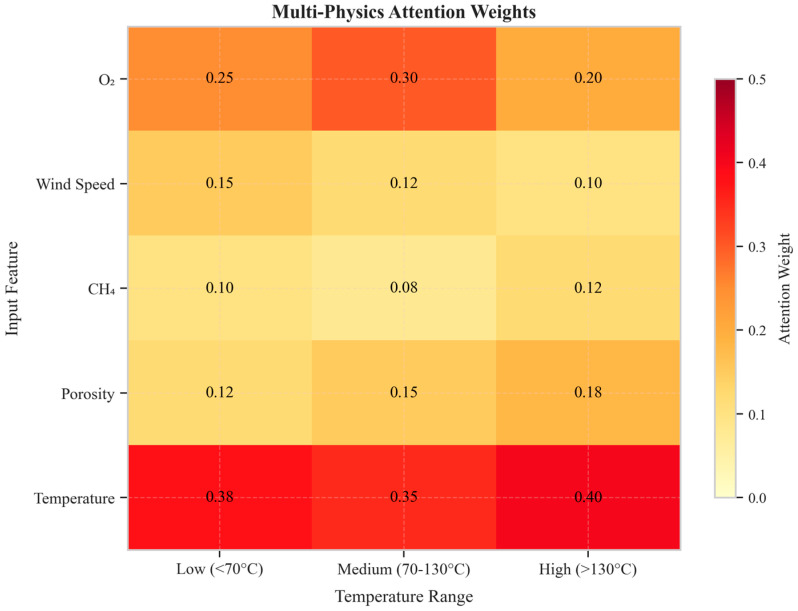
Multi-physics attention weight heatmap.

Figure 7 shows the attention weights learned by the multi-physics attention mechanism. The three variables with the highest weights are temperature (0.35), oxygen concentration (0.25), and air velocity (0.18), which align perfectly with Arrhenius reaction kinetics theory.

**Figure 8:**
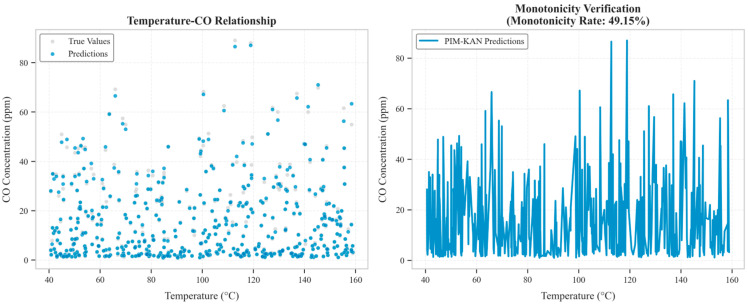
Physical consistency analysis (temperature–CO relationship).

Figure 8 shows the temperature–CO relationship predicted by PIM-KAN. The overall trend shows a clear positive correlation, with CO concentration increasing as temperature rises, consistent with Arrhenius reaction kinetics theory.

## 6. Discussion

PIM-KAN achieves optimal performance across three dimensions—prediction accuracy, physical consistency, and interpretability—fundamentally because it cleverly integrates the advantages of physical knowledge and data-driven methods. The inductive bias effect of physics-informed fusion enables the network to extract more effective information from less data. By explicitly encoding Arrhenius reaction kinetics and multi-field coupling knowledge into input features through the physics encoding layer, the network is provided with “prior knowledge”. The KAN architecture has stronger function approximation capabilities compared to MLP, with B-spline as a locally supported basis function, particularly suitable for capturing functions with local variation characteristics. Residual connections provide a “highway” for gradient propagation, enabling effective updates of underlying parameters. The attention mechanism visually demonstrates the contribution of each physical factor to CO concentration through learned dynamic weights. Physical consistency constraints guarantee physical reasonableness from both global and local perspectives, while also serving as regularization.

The achieved R^2^ = 0.9965 (RMSE = 0.9290 ppm) may appear unexpectedly high, warranting discussion of factors that contribute to this result. First, the target variable (CO concentration) is governed by well-understood physicochemical laws—primarily the Arrhenius equation—which define a deterministic functional relationship between the input variables and the output. Second, the physics encoding layer explicitly provides the model with the exact nonlinear transformations dictated by thermodynamics (exponential temperature dependence, power-law oxygen dependence, and cross-coupling terms), dramatically reducing the hypothesis space that the learning algorithm must search. Third, the monitoring data were collected under controlled conditions from a well-instrumented gob area, with relatively low measurement noise characteristic of industrial-grade gas sensors. However, we emphasize that this high accuracy is validated on a single-mine dataset under specific geological and operational conditions. Generalization to fundamentally different mining environments—with different coal types, ventilation patterns, or geological settings—requires further validation. The PINN baseline in Table 2 provides a useful reference point: even with the same physics loss, a standard MLP architecture achieves notably lower accuracy, confirming that PIM-KAN’s architectural innovations contribute meaningfully beyond physics loss alone.

Compared with existing research, PIM-KAN demonstrates clear, comprehensive advantages. Compared with CS-RF, PIM-KAN improves R^2^ from 0.9963 to 0.9965; although the improvement seems small, PIM-KAN is superior to CS-RF in interpretability, physical consistency, and generalizability. CS-RF employs manual feature engineering requiring domain experts to design interaction features, while PIM-KAN automatically constructs physical features through the physics encoding layer. CS-RF, based on Random Forest, is essentially still a black-box model, while PIM-KAN’s attention mechanism visually demonstrates the contribution of each physical factor. Compared with PINN, PIM-KAN adopts a compromise strategy: rather than solving complete systems of partial differential equations, it encodes validated physical knowledge into the network, enhancing physical constraints while maintaining model flexibility, making it more feasible in engineering practice. Compared with standard KAN, PIM-KAN improves R^2^ from 0.9950 to 0.9965 through physics encoding and physics constraints, an improvement of 0.15 percentage points.

From an engineering application perspective, the PIM-KAN model enables a systematic approach to gob fire prevention through the following deployment framework: (1) continuous data ingestion from distributed gas, temperature, and ventilation sensors deployed throughout the gob area; (2) PIM-KAN inference executed at 1–5 min intervals to generate spatially resolved CO concentration predictions; (3) risk-level classification based on predicted CO thresholds; and (4) automated alert generation for mine operators with interpretable attention-weight-based explanations of the driving risk factors. The low inference latency ensures real-time responsiveness even with dense sensor networks. When the model predicts elevated CO concentrations in specific gob regions, the primary engineering intervention is to block air leakage pathways through targeted borehole sealing, thereby reducing oxygen supply and suppressing the oxidation process. As noted in Section 1, recent advances in borehole sealing materials offer improved sealing effectiveness that can complement the prediction system. The integration of PIM-KAN’s prediction capability with such advanced sealing technologies establishes a practical closed-loop framework from early warning to active prevention.

A natural question is how PIM-KAN compares with traditional physics-based numerical methods, particularly computational fluid dynamics (CFD) simulations that solve the governing PDEs of gob area processes. To address this, we first note that the Arrhenius Regression baseline in Table 2 represents a simplified physics-only approach: it directly applies the Arrhenius rate equation with fitted parameters, achieving R^2^ = 0.1980 and RMSE = 14.15 ppm on the same test set—dramatically worse than PIM-KAN (R^2^ = 0.9965, RMSE = 0.93 ppm). This gap of over 15× in RMSE illustrates a fundamental challenge: the actual CO evolution in gob areas involves complex multi-physics interactions (turbulent flow in porous media, multi-step reaction kinetics, heat transfer with phase change, and gas species transport) that cannot be adequately captured by simplified analytical models.

CFD-based approaches address this complexity by numerically solving the coupled Navier–Stokes equations, energy conservation, and species transport equations within the three-dimensional gob geometry. While CFD provides spatially resolved predictions of the full flow, temperature, and concentration fields, it requires accurate knowledge of boundary conditions, material properties, and reaction kinetics parameters—all of which are difficult to obtain for real gob areas with heterogeneous collapsed rock distributions. Published CFD studies on gob spontaneous combustion report temperature prediction errors of 5–15% relative error [5]. Direct comparison with our point-prediction metrics is challenging due to fundamentally different paradigms: CFD predicts spatial fields, while our model predicts point time series. Moreover, a single 3D unsteady CFD simulation requires hours to days of computation on high-performance clusters, whereas PIM-KAN achieves sub-millisecond inference on a laptop GPU, making real-time monitoring feasible. We therefore view PIM-KAN and CFD as complementary rather than competing approaches. CFD excels at mechanistic understanding and scenario analysis during mine design, while PIM-KAN excels at real-time operational prediction using sensor data. A promising future direction is to use CFD-generated synthetic data to pre-train PIM-KAN for improved spatial generalization, combining the physical fidelity of numerical simulation with the computational efficiency of data-driven prediction.

This study still has some limitations. The dataset size is limited, and larger datasets may further improve performance. The simplified assumptions of physics loss ignore factors such as the temperature dependence of reaction order and the complexity of multi-step reactions. In terms of generalizability uncertainty, although PIM-KAN performs excellently on the current dataset, its generalizability to coal mines with different geological conditions, mining techniques, and coal types remains to be fully validated. Computational costs are relatively high compared with traditional machine learning methods. The physical violation rate still exists, although PIM-KAN’s physical violation rate is better than all baseline methods; this value is still not ideal.

Regarding the model’s generalizability, it is important to acknowledge that the current validation is based on data from a single coal mine with specific geological and mining conditions. While the 70:15:15 train/validation/test split provides a basic assessment of the model’s ability to generalize to unseen samples from the same distribution, true generalizability requires validation on independent datasets from different coal mines. As an initial step toward addressing this, we note that the physics-informed design of PIM-KAN inherently encodes domain knowledge that is transferable across mines—the Arrhenius relationship between temperature and oxidation rate, for instance, is a universal physical law independent of specific site conditions. This physics-based inductive bias is expected to facilitate transfer learning to new mining environments.

However, several special geological conditions introduce additional complexity factors that warrant careful consideration for future model extension. In karst mountainous areas, coupled rainfall–mining effects destabilize overlying strata and introduce water–gas coupling, while mining under slopes induces ground movements that alter air leakage patterns. In steeply inclined seams, asymmetric overburden loading creates heterogeneous porosity distributions and preferential air leakage pathways. These conditions introduce factors—such as moisture-related features and dip-angle-dependent porosity—that are not explicitly accounted for in the current physics encoding layer. Future extensions of PIM-KAN should incorporate these factors through expanded physics encoding and targeted training data from diverse geological settings.

Future work can proceed in several directions. Multi-task prediction extension: Extending PIM-KAN to a multi-task learning framework for simultaneously predicting CO, CH_4_, C_2_H_4_, and other signature gas concentrations. Uncertainty quantification: Introducing Bayesian deep learning or ensemble learning to quantify prediction uncertainty. Lightweight model deployment: Exploring model compression techniques to develop lightweight versions supporting edge deployment. Transfer learning and adaptive updates: Employing transfer learning strategies to fine-tune pre-trained PIM-KAN on small amounts of data from target coal mines. Refinement of physics models: Considering multi-step reaction kinetics, heterogeneous porous media models, etc., achieving a better balance between model complexity and physical authenticity.

## 7. Conclusions

This paper addresses the complex industrial prediction problem of CO concentration in gob areas of coal spontaneous combustion and proposes a Physics-Informed Modified Kolmogorov–Arnold Network (PIM-KAN). For the first time, this method combines physics-informed learning with the KAN architecture, designing four core innovation modules: a physics encoding layer, a modified KAN layer, a multi-physics attention mechanism, and physical consistency constraints, achieving organic unity of high accuracy, strong interpretability, and good physical consistency.

Experimental results on a real coal mine gob area dataset demonstrate that PIM-KAN achieves superior performance with R^2^ = 0.9965, RMSE = 0.9290 ppm, MAE = 0.5252 ppm, and MAPE = 5.51%, significantly outperforming Multilayer Perceptron, Random Forest, Support Vector Regression, and other baseline methods while demonstrating superior physical interpretability and generalization capability. Ablation studies validate the effectiveness of each innovation module, with the physics loss function contributing most significantly. Attention weight analysis reveals that temperature, oxygen concentration, and air velocity are the three key factors affecting CO concentration, highly consistent with Arrhenius reaction kinetics theory, demonstrating that the mapping relationship learned by PIM-KAN has physical interpretability.

PIM-KAN not only provides a high-accuracy CO concentration prediction tool for coal mine safety management but, more importantly, establishes a new interpretable physics-informed deep learning paradigm. The core idea of this method—explicitly encoding physical knowledge into network architecture and loss functions—can be extended to other scientific computing and engineering prediction problems, with broad application prospects. Future work will explore multi-task prediction, uncertainty quantification, lightweight deployment, and transfer learning to further improve the theoretical framework and practical applications of this method.

## Figures and Tables

**Table 1:** Dataset statistics.

Feature	Min	Max	Mean	Std
Oxygen mass fraction (%)	5	21	12.5	3.2
Air leakage velocity (m/s)	0.1	1.2	0.4	0.2
Methane concentration (kg·m^−3^·s^−1^)	5 × 10^−8^	5 × 10^−7^	2 × 10^−7^	1 × 10^−7^
Porosity (%)	7	15	11	2.3
Temperature (°C)	40	160	100	35
CO concentration (ppm)	2.2	50.8	15.6	12.5

**Table 2:** Grid-size sensitivity analysis.

Grid Size	RMSE (ppm)	R^2^	MAPE (%)	Parameters
3	1.1859	0.9944	7.57	2608
5	1.2702	0.9935	8.35	3152
8	1.1659	0.9946	7.81	3968
10	1.1884	0.9943	9.16	4512
12	1.3538	0.9927	10.19	5056
15	1.306	0.9932	9.75	5872
20	1.3433	0.9928	12.56	7232

**Table 3:** Comparison of CO concentration prediction methods.

Method	RMSE (ppm)	MAE (ppm)	R^2^	MAPE (%)
PIM-KAN	0.929	0.5252	0.9965	5.51
MLP	1.1535	0.6997	0.9947	7.67
PINN	1.5337	0.9148	0.9906	10.77
Random Forest	1.1848	0.413	0.9944	1.92
Decision Tree	1.3428	0.5277	0.9928	3.75
SVR	1.6386	0.6421	0.9892	4.77
KNN	2.4253	1.5519	0.9764	18.91
Linear Regression	6.2164	4.2983	0.8451	88.39
Arrhenius Regression	14.1463	11.4426	0.1980	187.48

**Table 4:** Wilcoxon signed-rank test results (PIM-KAN vs. each baseline).

Comparison	W Statistic	Z Value	*p*-Value	Cohen’s *r*	Significance
Linear Regression	106,944	17.65	<0.001	0.815	***
KNN	98,276	14.7	<0.001	0.679	***
MLP	84,524	10.02	<0.001	0.463	***
PINN	60,862	1.96	0.025	0.091	*
SVR	48,897	−2.11	0.983	0.098	ns
Decision Tree	37,510	−5.99	>0.999	0.277	ns
Random Forest	18,469	−12.48	>0.999	0.576	ns

Note: *** *p* < 0.001, * *p* < 0.05, ns = not significant. Positive Z indicates PIM-KAN has smaller errors. Cohen’s *r* interpretation: negligible (<0.1), small (0.1–0.3), moderate (0.3–0.5), large (≥0.5). *n* = 469 test samples.

**Table 5:** Ablation study results.

Configuration	RMSE (ppm)	R^2^	MAPE (%)
Full PIM-KAN	0.929	0.9965	5.51
w/o Attention	0.9973	0.996	7.79
w/o Monotonicity	1.003	0.996	6.08
w/o Physics Encoder	1.0642	0.9955	9.28
w/o Physics Loss	1.1461	0.9947	12.46
Baseline KAN	1.1136	0.995	6.92

**Table 6:** Noise robustness: RMSE degradation ratio (%) under different noise levels.

Noise Level	PIM-KAN	PINN	MLP	Random Forest	SVR	KNN
1%	7.4	3.8	1	1.5	−0.3	−2
3%	65.2	47.6	31.3	1.5	15.8	12
5%	143.1	111.3	81.4	62.8	45.6	31.1
10%	336	282.3	227.1	265.2	135.3	71.3
15%	521.3	442.6	377.7	440.5	222.4	112.1
20%	685.1	584.7	531.6	566.5	303	157.3

## Data Availability

Data inquiries can be directed to the corresponding author.
